# Feelings of Hopelessness in Midlife and Cognitive Health in Later Life: A Prospective Population-Based Cohort Study

**DOI:** 10.1371/journal.pone.0140261

**Published:** 2015-10-13

**Authors:** Krister Håkansson, Hilkka Soininen, Bengt Winblad, Miia Kivipelto

**Affiliations:** 1 Center for Alzheimer Research, Division of Neurogeriatrics, Department of NVS, Karolinska Institutet, Stockholm, Sweden; 2 Aging Research Center (ARC), Department of NVS, Karolinska Institutet and Stockholm University, Stockholm, Sweden; 3 Department of Neurology, Kuopio University Hospital and University of Eastern Finland, Kuopio, Finland; University Of São Paulo, BRAZIL

## Abstract

**Background:**

Several studies have found depression and depressive feelings to be associated with subsequent dementia. As dementias typically have a long preclinical development phase, it has been difficult to determine whether depression and depressive feelings reflect a concurrent underlying dementia disease, rather than playing a causative role. Our aim was to investigate hopelessness, one dimension of depressive feelings, and evaluate the likelihood of a prodromal versus a causative role of hopelessness feelings in dementia development.

**Methods:**

We invited a random sample of 2000 survivors from a representative population in Eastern Finland, originally investigated in midlife between 1972 and 1987, for re-examination an average of 21 years later. The age of the 1449 persons who accepted the invitation was between 39 and 64 years (mean 50.4 years) in midlife and between 65 and 80 (mean 71.3) at follow-up. To measure feelings of hopelessness in midlife and at follow-up, the participants indicated their level of agreement to two statements about their own possible future. We used logistic regression to investigate the association between the combined scores from these two items and cognitive health at follow-up, while adjusting for several health and life-style variables from midlife and for apolipoprotein E4 (ApoE4) status, depression and hopelessness feelings at follow-up. We compared the associations with late-life cognitive health when feelings of hopelessness were either measured in midlife or at the follow-up. In addition we analyzed the changes in hopelessness scores from midlife to follow-up in participants who were either cognitively healthy or impaired at follow-up.

**Results:**

We found higher levels of hopelessness *in midlife*, but not at follow-up, to be associated with cognitive impairment at follow-up; the adjusted odds ratio for each step of the five-level hopelessness scale was 1.30 (95% confidence interval 1.11–1.51) for any cognitive impairment and 1.37 (1.05–1.78) for Alzheimer’s disease. These associations remained significant also after the final adjustments for depressive feelings and for hopelessness at follow-up. The individual changes in hopelessness scores between midlife and follow-up were not systematically related to cognitive health at the follow-up.

**Conclusion:**

Our results suggest that feelings of hopelessness already in midlife may have long-term implications for cognitive health and increase the risk of Alzheimer’s disease in later life.

## Introduction

Alzheimer’s disease, the most common type of dementia, has a progressive nature with a demonstrated preclinical cognitive decline by up to twelve years [1]. Several longitudinal studies have reported an association between depression and dementia [2–11], including depressive feelings below criteria for clinical depression [6,8,9,12]. Two prospective studies with repeated measures during the prodromal period of Alzheimer’s disease found no [12], or a “barely perceptible” [13] increase in depressive feelings, suggesting an association that does not reflect reverse causation. A third study with a similar design found an association between depressive feelings, both at midlife and in later life, and vascular dementia. For Alzheimer’s disease, the same study reported an association only with depressive symptoms in later life, interpreted by the authors as evidence of a prodromal relation [14].

Hopelessness has been suggested as a central dimension of depression [15], as well as a marker of anxiety [16], and a predictor of clinical depression [16]. Others have suggested a specific relevance for hopelessness—apart from global depression—in predicting various health outcomes: In the 1970s, Aaron Beck developed the Hopelessness Scale [15], a 20-item forced choice (yes-no) questionnaire, with the original aim of predicting suicidal ideation and eventual suicide better than the Beck Depression Index (BDI) [17]. Several studies do indicate that hopelessness is an equal or stronger predictor of suicide than depression [18,19], and one study found the single pessimism item of the Beck Depression scale to differentiate suicide completers from non-completers, whereas the scale as a whole did not [17]. Several studies have also found hopelessness to be an equal or stronger predictor of mortality and morbidity than global depression [20], even at moderate levels [21,22].

We are unaware of any prospective studies on the association between feelings of hopelessness and cognitive impairment. One study has reported a protective association between the somewhat related concept *purpose in life* and Alzheimer’s disease [23], while a recent study found *apathy* to be associated with incident mild cognitive impairment [24]. In this study we were able to compare the significance of hopelessness in midlife versus later life for cognitive impairment in later life and to relate these associations both to depressive symptoms and to the main genetic risk factor for Alzheimer’s disease, the apolipoprotein E4 allele (ApoE4).

## Methods

### Study design overview

We used a population-based cohort design with participants from two regions in Eastern Finland. They were first examined in midlife (mean age 50.4 years, SD 6.0) and then again averagely more than two decades later (mean follow-up time 20.9 years, SD 4.9). The main predictor variable was feelings of hopelessness, measured both at baseline and follow-up. The main outcome variable was cognitive status at follow-up, established through careful diagnostic procedures to differentiate between normal aging, mild cognitive impairment and different types of dementia. Adjustments were made for a number of variables measured at baseline and also to gender, ApoE4 status and to age and depressive symptoms at follow-up.

### Subjects

The study was based on the Cardiovascular risk factors, Aging and Dementia (CAIDE) Study. Participants were the survivors of four separate, independent, population-based random samples, originally examined to assess cardiovascular risk factors within the North Karelia Project and the FINMONICA study. The four samples were randomly drawn from the population register of two regions in Eastern Finland, the North Karelia and Kuopio regions, and comprised a total of 30 078 participants, aged 30–59 [25]. Participation rates in the initial investigations (either 1972, 1977, 1982 or 1987) were 82–90% [25].

By the end of 1997, we selected a random sample of 2000 individuals from the original population among those who still lived in or nearby the cities of Kuopio or Joensuu and who had then reached an age between 64 and 79 years.

**Fig 1:**
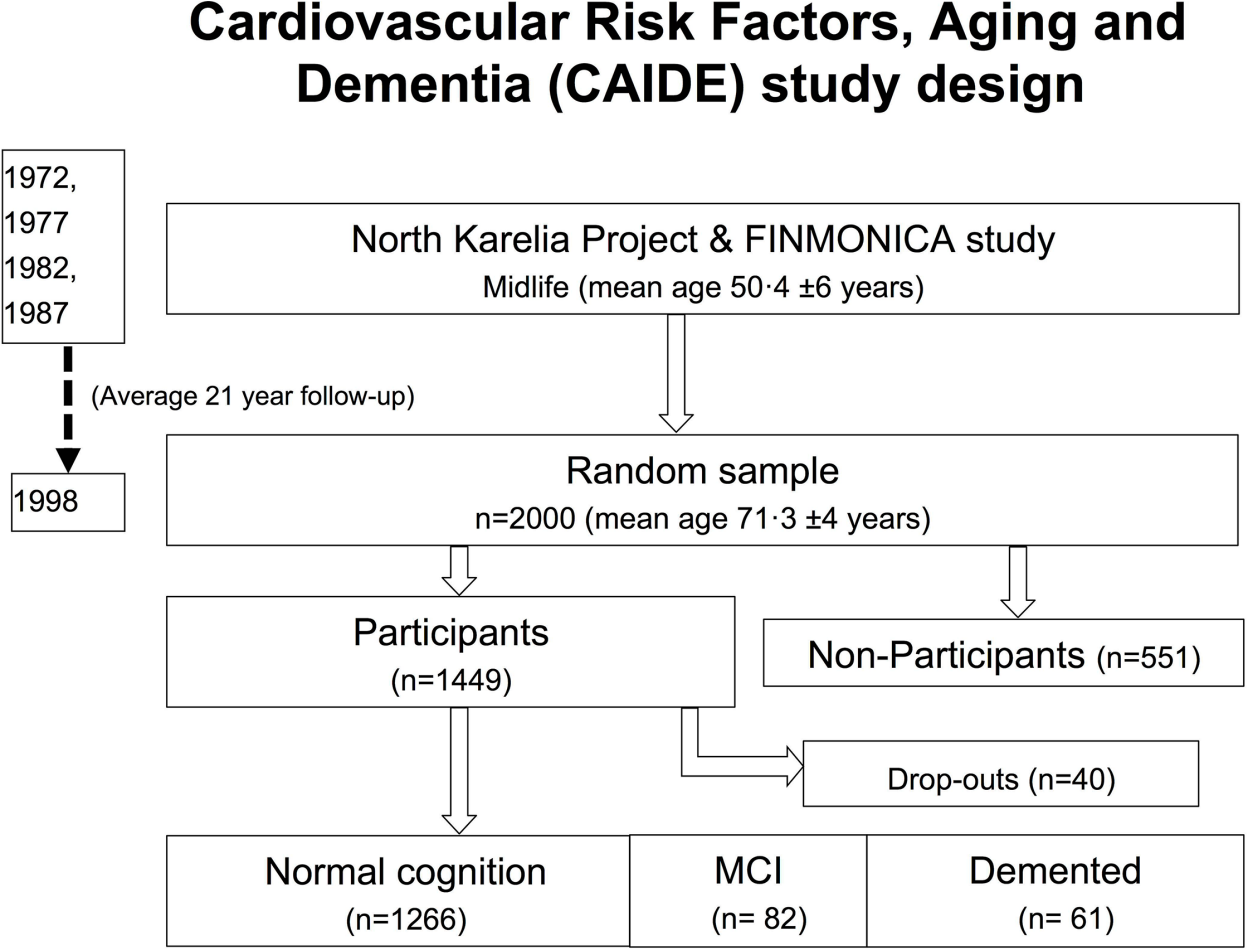
Flowchart of the study design. A random sample of 2000 of the participants from the North Karelia and FINMOCA study, originally investigated in either 1972, 1977, 1982 or 1987, were invited for a reinvestigation in 1998. For health reasons or other reasons, 551 (27%) did not accept the invitation. During the measurements, 40 persons dropped out, leaving 1409 to be included in this study.

When invited for a re-examination during the following year, 1449 (73%) of these persons agreed to participate. Their mean age was 71.3 (SD 4.9) years. Of those who after initial screening emerged as possible cases of cognitive impairment, 40 participants dropped out, either as a result of refusal (33 persons), poor health (five persons) or death (two persons), leaving 1409 for the final analysis (see flow chart, Fig 1).

After complete description of the study to the participants, we obtained written informed consent from all of them.

### Midlife measurements

The survey methods we used at baseline (midlife) complied with the WHO MONICA protocol, described in more detail elsewhere [25]. In brief, the baseline survey included a self-administered questionnaire on health behavior, health status, marital status, socio-economic status, psychosocial factors (including feelings of hopelessness) and medical history. Nurses trained for the survey ensured that the questionnaires were fully completed and understood. We measured participants’ blood pressure, serum cholesterol levels, height and weight, and determined their BMI (weight (kg)/(height (m)^2^). The two hopelessness items were identical to the questions used in previous studies on hopelessness and mortality [20,22]. Their exact wording was: "I feel that it is impossible to reach the goals I would like to strive for" and "The future seems to me to be hopeless, and I can't believe that things are changing for the better". A five-point Likert scale was used, originally coded as 0 = absolutely agree; 1 = somewhat agree; 2 = cannot say; 3 = somewhat disagree; or 4 = absolutely disagree. In the data analysis we reversed the scores in order for higher scores to reflect a higher degree of hopelessness. No composite measure of depressive symptoms was used at baseline.

### Measurements in later life

During the re-examination in 1998, we used the same survey methods as in midlife. In addition, we identified participants apolipoprotein genotype by using polymerase chain reaction and *Hhal* digestion, as described by Tsukamoto et al [26]. We assessed cognitive status through a three-step protocol for the diagnosis of dementia: a screening phase, a clinical phase, and a differential diagnostic phase. The 294 subjects who scored 24 or less on the Mini-Mental State Examination (MMSE) [27] underwent thorough neurological, cardiovascular and neuropsychological assessments. To diagnose dementia, we used the criteria from the Diagnostic and Statistical Manual of Mental Disorders, 4^th^ edition. The diagnostic criteria for Alzheimer’s disease were fulfilled in 48 participants according to National Institute of Neurological and Communicative Disorders and Stroke and the Alzheimer’s Disease and Related Disorders Association Criteria [28]. The diagnosis was confirmed if magnetic resonance imaging showed generalized or medial temporal lobe atrophy, or both, but without indications of vascular dementia. We also found other types of dementia in 13 participants (vascular dementia, Parkinson’s disease dementia and alcohol dementia).

We diagnosed 82 persons with mild cognitive impairment, according to the criteria advised by the Mayo Clinic Alzheimer’s Disease Research Center [29]. The main criteria were objective impairment below 1.5 standard deviations of the age appropriate mean in either memory or one other area of cognitive functioning, along with memory complaints.

Feelings of hopelessness were estimated prior to the assessment of cognitive status and in the same way as at baseline. To estimate depressive feelings, we used The Center for Epidemiological Studies Depression scale (CES-D) [30].

### Statistical analyses

*Cognitive impairment* was first treated as an all-inclusive variable, comprising 143 persons diagnosed with either mild cognitive impairment or any type of dementia. For more detailed analysis, we also performed calculations with *mild cognitive impairment (MCI)* and *Alzheimer’s disease (AD)* as separate outcomes. In each of the three analyses, the 1266 subjects without any signs of cognitive impairment served as reference group.

The scores from the two questions on hopelessness were summed, resulting in a scale that was approximately normally distributed (skewness 0.23 and 0.17 at baseline and follow-up, respectively, and with a kurtosis of -0.25 and -0.52). In the first analysis this scale was entered as a five-level ordinal scale after collapsing categories with fewer than 10% of the observations (scores 0–1 and 5–8). After the transformation each scale level included between 16.5% and 26.6% of the participants. To facilitate comparison with other studies, additional analyses were based on a median split dichotomization of the original hopelessness scale.

We calculated a hopelessness change score for each participant by subtracting his or her follow-up score from the baseline score in order to investigate if participants with an elevated score at follow-up differed in cognitive health from those who were either stable or had a lower hopelessness score at follow-up.

We used logistic regression to analyze the associations of feelings of hopelessness, both at baseline and follow-up, with cognitive impairment at follow-up. Adjustments were made for a number of variables with a probable theoretical significance and/or that showed and association with the outcome variables (see Table 1) or with the predictor variable (see Table 2). We adjusted the associations in three steps with age, ApoE4 status, education and gender in the first model. In Model 2 we additionally adjusted for the following variables from midlife: systolic blood pressure, cholesterol, BMI, region of residence, physical strain at work, type of work (white versus blue collar), smoking and marital status (cohabitants versus non-cohabitants). To investigate if feelings of hopelessness would predict cognitive health independently of composite depressive feelings, we entered each participants CES-D score from follow-up in the last step of the logistic regression equation (Model 3). For the 219 persons (15.5%) without data on depression, CES-D scores were obtained by pooling estimates from multiple imputations.

**Table 1:** Characteristics of participants with and without cognitive impairment at follow-up.

Variables	Cognitively healthy (N = 1266)		Cognitively impaired (N = 143)		
**General Characteristics**					
	**N**	**%**	**N**	**%**	**p-value**
Gender, female	785	62.0	90	62.9	n.s.
ApoEε4 carriers with one or two ε4 alleles	427	34.5	62	45.3	0.01
Residence area in Kuopio vs Joensuu	600	47.4	90	63.6	<0.001
	**Mean**	**SD**	**Mean**	**SD**	**p-value**
Follow-up time (years)	20.9	5.0	21.1	4.6	n.s.
**Characteristics at baseline**					
	**Mean**	**SD**	**Mean**	**SD**	**p-value**
Age (years)	50.1	6.0	52.4	5.4	<0.001
Education (years)	8.84	3.5	6.79	2.5	<0.001
Systolic blood pressure (mm/Hg)	143	19	151	22	<0.001
Total cholesterol (mmol/l)	6.68	1.2	7.16	1.1	<0.001
Physical strain at work	1.91	0.89	2.07	0.98	.05
Commuting physical activity	2.73	1.6	2.62	1.6	n.s.
Leisure time activity	2.93	1.4	2.97	1.4	n.s.
Type of occupation (%with white collar jobs vs other occupations)	47.7	47.7	29.4	29.4	<0.001
BMI (kg/m2)	26.5	3.7	27.5	3.7	0.002
Feelings of hopelessness	3.02	1.8	3.83	1.6	<0.001
	**N**	**%**	**N**	**%**	**p-value**
Type of occupation (white collar jobs vs others)	604	47.7	42	29.4	<0.001
Smoker	548	43.3	57	39.9	n.s.
Living in a cohabitant relation (%)	1003	81.6	100	69.9	0.002
**Characteristics at follow-up**					
	**Mean**	**SD**	**Mean**	**SD**	**p-value**
Age (years)	71.0	3.9	73.5	4.1	<0.001
CES-D score	13.6	4.5	15.7	6.7	<0.001
Feelings of hopelessness	3.04	1.8	3.81	1.9	<0.001
MMS score	26.3	1.9	21.9	2.5	<0.001

The two-tailed P-values are derived from Students t-test for continuous variables and χ2 for nominal variables.

**Table 2:** Characteristics of participants with low and high levels of hopelessness in midlife.

Variables	Low hopelessness (N = 785)		High hopelessness (N = 581)		
**General Characteristics**					
	**N**	**%**	**N**	**%**	**p-value**
Gender, female	465	59.2	374	64.4	n.s.
ApoEε4 carriers with one or two ε4 alleles	289	37.6	188	33.3	n.s.
Residence area in Kuopio vs Joensuu	375	47.8	296	50.9	n.s.
	**Mean**	**SD**	**Mean**	**SD**	**p-value**
Follow-up time (years)	21.0	4.8	21.0	4.8	n.s.
**Characteristics at baseline**					
	**Mean**	**SD**	**Mean**	**SD**	**p-value**
Age (years)	49.9	6.0	50.6	6.0	0.03
Education (years)	9.33	3.71	7.79	2.89	<0.001
Systolic blood pressure (mm/Hg)	143	20	144	19	n.s.
Total cholesterol (mmol/l)	6.67	1.22	6.79	1.19	0.06
Physical strain at work	1.88	0.83	2.01	0.99	0.006
Commuting physical activity	2.79	1.60	2.63	1.63	n.s.
Leisure time activity	2.92	1.30	2.97	1.51	n.s.
BMI (kg/m2)	26.2	3.5	27.0	3.8	<0.001
	**N**	**%**	**N**	**%**	**p-value**
Type of occupation (white collar jobs vs others)	54.8	54.8	34.4	34.4	<0.001
Living in cohabitant relation	82.0	82.0	79.0	79.0	n.s.
Smoker	42.9	42.9	45.4	45.4	n.s.
**Characteristics at follow-up**					
	**Mean**	**SD**	**Mean**	**SD**	**p-value**
Age (years)	70.9	4.0	71.6	4.0	0.002
CES-D score	12.7	4.4	15.4	5.7	<0.001
Feelings of hopelessness	2.51	1.7	3.93	1.7	<0.001
MMS score	26.2	2.1	25.4	2.6	<0.001
	**N**	**%**	**N**	**%**	**p-value**
Cognitive impairment (%)	*53*	6.8	86	14.8	<0.001

The two-tailed P-values were derived from Students t-test for continuous variables and χ2 for nominal variables.

We also compared carriers and non-carriers of the ApoE4 allele to see if the associations between hopelessness and cognitive impairment differed between these subgroups.

To address the issue of a prodromal versus a causal relationship we compared the associations of midlife versus later-life levels of hopelessness with cognitive impairment. If feelings of hopelessness would be mainly a consequence of cognitive impairment, rather than a predictor of it, we expected levels of hopelessness, measured shortly before diagnosis, to show the stronger association.

The mean age of the participants at baseline was 50.4 years, ranging from 39 to 64 years. To evaluate the effect of age at baseline, we used median age at baseline as cut point to form a younger group aged 39–50 (mean 45.4, SD 3.0) and an older group aged 50–65 (mean 55.5, SD 3.5). Separate logistic regression calculations were performed for each of these age-based subgroups. These analyses were also used to further evaluate the risk of reverse causation with the hypothesis that if anyone were affected by early sub-clinical cognitive impairment already at baseline, they would more likely belong to the relatively older subgroup.

All analyses were performed in SPSS v22 for Mac OS X by the main author. In all analyses we used a p value of 0·05 (two-sided) as criterion for statistical significance.

### Ethics

The local Ethics Committees in Finland (UEF Board on Research Ethics, Kuopio) and in Sweden (Regional Ethical Research Board, Stockholm) approved the study, including the procedure to obtain written informed consent from all participants.

## Results

### Hopelessness in midlife and cognitive impairment in later life

The original association between hopelessness and cognitive impairment shown in Table 1 remained significant also as estimated through logistic regressions, both when hopelessness was entered as continuous and as categorical (first part of Table 3). When entered as continuous, the adjusted odds ratio for any cognitive impairment was 1.30 (1.11–1.51) for each step of the five-level scale of hopelessness (first part of Table 3) and 1.26 (1.07–1.50) also after adjustments for depression and hopelessness at follow-up. With hopelessness as a dichotomous variable (participants with scores 4–8 versus 0–3), the adjusted odds ratio was 2.90 (1.4–5.9) with Alzheimer’s disease as outcome (first part of Table 3) and 2.46 (1.1–5.3) after the additional adjustments for depression and hopelessness at follow-up.

**Table 3:** Association between feelings of hopelessness and cognitive impairment in later life.

	Any cognitive impairment Analysis based on N = 1305 (n = 132 with cognitive impairment)			Mild Cognitive Impairment (MCI) Analysis based on N = 1252 (n = 79 with MCI)			Alzheimer’s Disease (AD) Analysis based on N = 1212 (n = 45 with AD)		
	OR	95% CI	p	OR	95% CI	p	OR	95% CI	p
Associations from midlife									
**Hopelessness as continuous variable (five levels)**									
Crude value*	1.45	1.26–1.67	< .001	1.42	1.20–1.69	< .001	1.48	1.17–1.88	.001
Model 1**	1.34	1.15–1.55	< .001	1.30	1.08–1.56	.005	1.37	1.07–1.77	.013
Model 2***	1.30	1.11–1.51	.001	1.28	1.06–1.54	.011	1.37	1.05–1.78	.020
Model 3****	1.24	1.06–1.45	.009	1.26	1.04–1.53	.020	1.25	0.95–1.64	.119
Model 4*****	1.26	1.07–1.50	.007	1.29	1.05–1.57	.014	1.27	0.94–1.72	.112
**Hopelessness dichotomized according to median split (scores 0–3 vs 4–8)**									
Crude value*	2.56	1.8–3.7	< .001	2.34	1.5–3.7	< .001	3.24	1.7–6.2	< .001
Model 1**	2.11	1.4–3.1	< .001	1.90	1.2–3.1	.008	2.84	1.4–5.6	.003
Model 2***	1.98	1.3–3.0	.001	1.81	1.1–3.0	.018	2.90	1.4–5.9	.009
Model 3****	1.78	1.2–2.7	.007	1.75	1.1–2.9	.030	2.35	1.1–4.9	.022
Model 4*****	1.84	1.2–2.8	.006	1.83	1.1–3.1	.024	2.46	1.1–5.3	.021
Associations in later life									
**Hopelessness as continuous variable (five levels)**									
Crude value*	1.36	1.17–1.57	< .001	1.24	1.04–1.48	.015	1.64	1.23–2.18	.001
Model 1**	1.28	1.10–1.49	.002	1.07	0.89–1.30	.457	1.51	1.12–2.04	.006
Model 2***	1.13	0.96–1.33	.152	1.04	0.85–1.26	.729	1.38	1.00–1.90	.051
Model 3****	1.03	0.86–1.24	.858	1.01	0.82–1.24	.928	1.08	0.74–1.57	.683
**Hopelessness dichotomized according to median split (scores 0–3 vs 4–8)**									
Crude value*	2.15	1.4–3.2	< .001	1.83	1.1–3.0	.016	2.76	1.3–5.8	.007
Model 1**	1.41	0.9–2.2	.113	1.22	0.7–2.0	.456	1.65	0.8–3.6	.212
Model 2***	1.27	0.8–2.0	.288	1.09	0.6–1.8	.753	1.60	0.7–3.7	.267
Model 3****	1.03	0.6–1.7	.726	1.03	0.6–1.8	.930	0.83	0.3–2.2	.712

* **Crude values** are odds ratios (OR) and 95% confidence intervals (CI) from logistic regression without adjustments.

** **Model 1:** with adjustments for age at follow-up, ApoEε4, gender, and years of education.

*** **Model 2:** with adjustments for the following variables from midlife: systolic blood pressure, cholesterol, BMI, region of residence, physical strain at work, type of work (white vs blue collar), smoking and marital status (cohabitants vs non-cohabitants).

**** **Model 3:** with additional adjustment for depressive feelings at follow-up, measured through CES-D.

***** **Model 4:** with additional adjustment for hopelessness at follow-up in the association calculations from midlife.

*Participants without data on hopelessness were excluded from the analyses with hopelessness as predictor*.

When we added depressive feelings to the previous adjustments, the hopelessness association weakened by averagely 24%, but still remained statistically significant in five out of six calculations (first part of Table 3).

When the sample was divided into a relatively older and younger subgroup with reference to age at baseline, the fully adjusted odds ratios for any cognitive impairment were 1.19 (1.03–1.38) for the older and 1.31 (1.09–1.57) for the younger subgroup.

### Hopelessness and cognitive impairment in later life

Also when measured at follow-up, feelings of hopelessness were significantly more pronounced among participants who were to be diagnosed as cognitively impaired (see Table 1 and also the corresponding crude (non-adjusted) odds ratios in the lower part of Table 3). This association was however largely eradicated when adjustments were made and only reached borderline statistical significance in one out of six comparisons before the final adjustment for depression (lower part of Table 3). The only remaining significant association was with hopelessness as continuous variable and with Alzheimer’s disease as outcome. We tried several alternative calculations to test the relative weakness of the adjusted associations between feelings of hopelessness in later life and cognitive health, including imputation of hopelessness in later life to maximize N, using adjustment with non-imputed CES-D data, and substituting adjustment variables from midlife with corresponding variables from later life (marital status, BMI, blood pressure, cholesterol, physical exercise, and smoking). None of these alternative calculations strengthened the original associations.

### Differences in levels of hopelessness between baseline and follow-up

**Fig 2:**
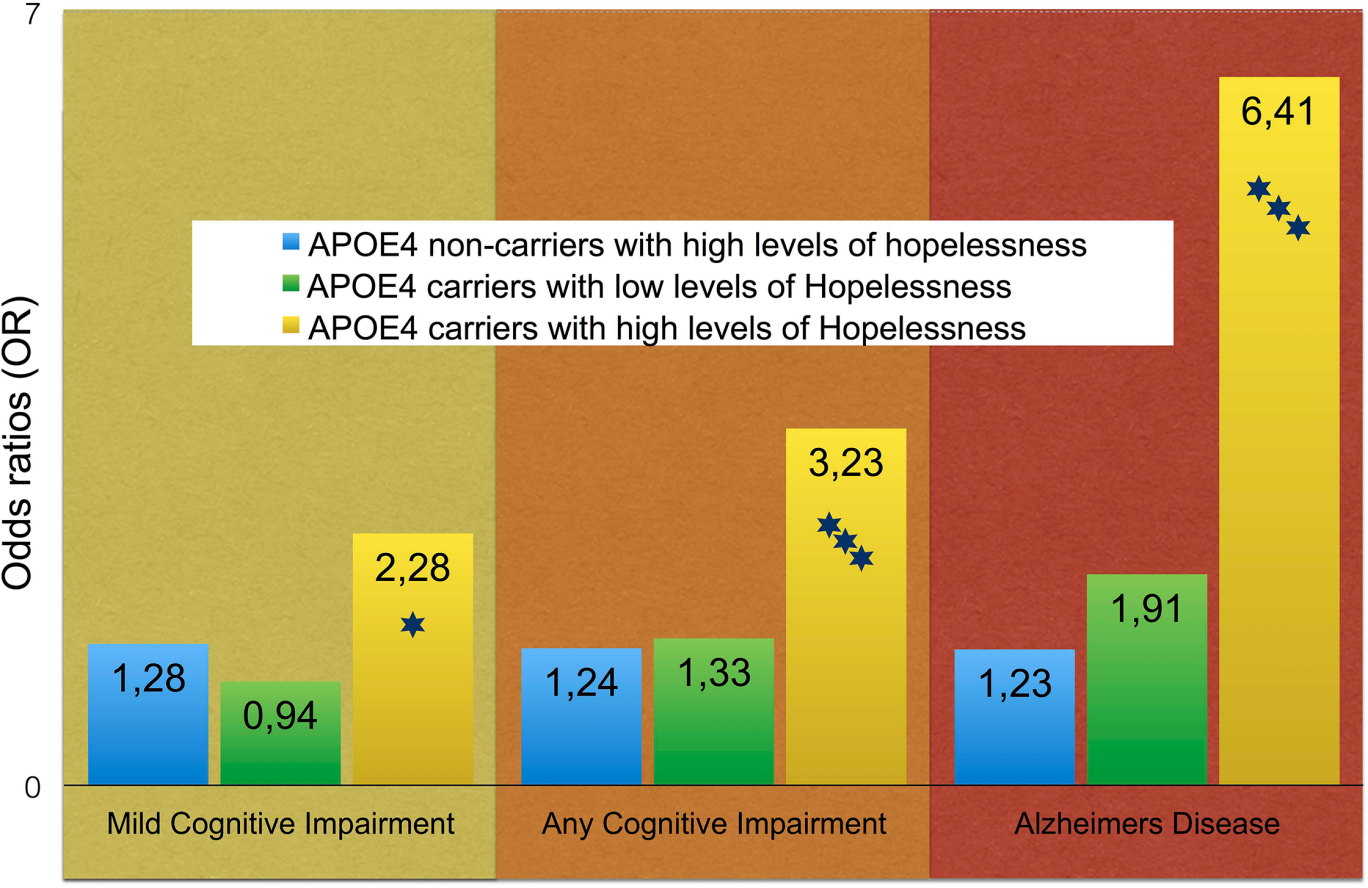
Levels of hopelessness at baseline and follow-up for the different outcome groups. Changes in hopelessness scores from baseline until follow up for those who at follow-up were either without cognitive impairment or were diagnosed with any cognitive impairment, mild cognitive impairment or Alzheimer’s disease. No changes from baseline to follow-up were statistically significant within any of the outcome categories (Student t-test for independent samples). All differences between the non-impaired group and any of the cognitive impairment groups were statistically significant, both at baseline and at follow-up (* = p≤0.05, ** = p≤0.01, *** = p≤0.001 as indicated by Students t-test for paired samples). The graph is based on scores from participants with measurements of hopelessness both at baseline and follow-up (N = 1246).

We found no significant differences in scores of hopelessness between baseline and follow-up *within* any of the outcome groups, including within the cognitively healthy reference group. The pattern of these results is summarized in Fig 2. As indicated in that figure, only the group that developed Alzheimer’s disease showed a slight increase in feelings of hopelessness (+0.35 (SD 1.64) scale units), but this difference between midlife and follow-up did not come close to statistical significance (p = 0.39, Students t-test for paired samples).

To further investigate the significance of change in hopelessness feelings from midlife to later life, we calculated individual change scores (each persons score in later life subtracted by his or her midlife score). The change scores showed no association to cognitive health status at follow-up as tested through Students t-tests and also not through Chi-2 after categorizing the participants into three categories of no or almost no change (61% of the participants with ≤1 in score difference) and participants who had either an increase or decrease in hopelessness score of more than one point. We also tried with logistic regression in order to adjust the change scores in the same way as in the main calculations, but none of the produced odds ratios came close to statistical significance.

### Associations among carriers and non-carriers of ApoE4

**Fig 3:**
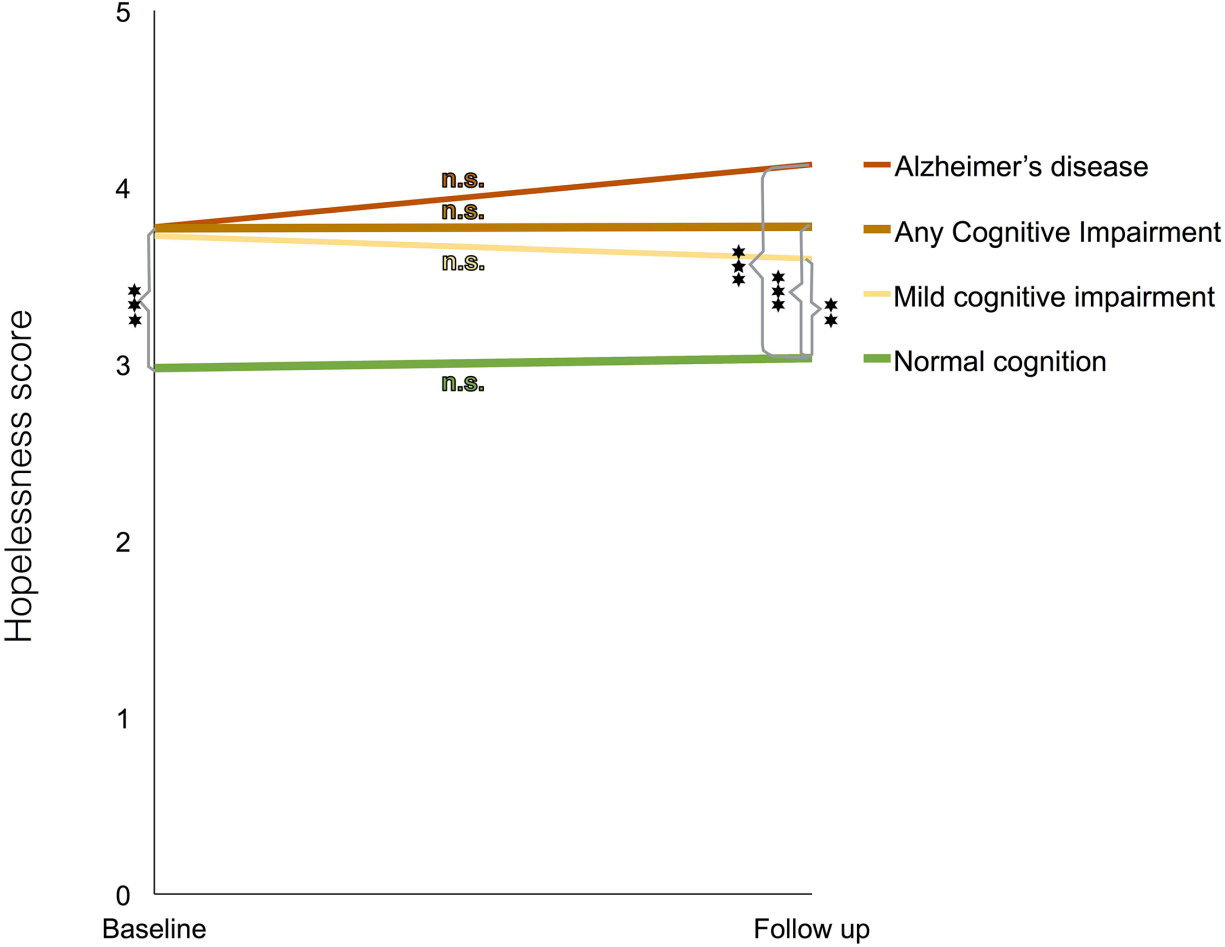
Hopelessness and cognitive impairment for ApoE4 carriers and non-carriers. Odds ratios from logistic regressions after adjustments for age, education, gender, BMI, blood pressure, cholesterol, residence area, occupation, physical activity, smoking, marital status, and depression. The risk is in comparison to ApoE4 non-carriers with low levels of hopelessness at midlife (OR = 1). Stars indicate the level of statistical significance (three levels <0.05, <0.01, <0.001).

When we subdivided the participants into ApoE4 carriers and non-carriers, the association between hopelessness and cognitive impairment was more pronounced among ApoE4 carriers, especially with Alzheimers disease as outcome. With non-carriers with low levels of hopelessness at midlife as reference, this subgroup had an adjusted odds ratio of 8.08 (3.1–21.1). After final adjustment also for depression, the odds ratio was 6.48 (2.4–17.5) (Fig 3).

### Participants and non-participants

To evaluate a possible participation bias we compared baseline data between the participants and the 551 non-participants. Non-participants had higher levels of hopelessness (3.43 versus 3.10, p < .001), fewer years of education (7.53 versus 8.63, p < .001), higher BMI (27.2 versus 26.5, p = .001), higher blood pressure (150 versus 144, p < .001), higher cholesterol levels (6.98 versus 6.73, p < .001), were somewhat older (72.3 versus 71.3, p < .001). They were also to a less extent cohabitant with a partner (72.7% versus 80.4%, p < .001) and white collar workers (33.5% versus 45.8, p < .001). According to linked register data they were also more likely to have a dementia diagnosis in 1998 (16.7% versus 7.8%, p = .005). There were no statistically significant differences in gender, smoking or any of the physical activity variables.

## Discussion

In this prospective population-based study, we found that feelings of hopelessness, measured already in midlife, predicted cognitive impairment an average of 21 years later. The results were statistically significant for all three cognitive impairment outcomes (any cognitive impairment, mild cognitive impairment, and Alzheimer’s disease) and consistent regardless of whether we used the continuous or the dichotomous hopelessness variable. The associations also remained significant after adjustments for several relevant variables, even after final adjustment for depressive feelings in later life.

On the other hand, the corresponding associations between feelings of hopelessness in later life and cognitive impairment did not hold after the same adjustments and in most cases lost statistical significance even before adjustment for depressive feelings in the last step. This should mean that changes in hopelessness feelings from midlife to later life are not systematically related to the risk of cognitive impairment in later life, a conclusion we could confirm through calculations based on individual change scores.

The most straight-forward interpretation of this relatively higher midlife significance of hopelessness feelings could be that feelings of hopelessness are causally related to cognitive impairment—or that such feelings are a marker of some other early causal factor: As dementia is a progressive disease with a long subclinical phase, the time window for any causal/triggering factor would have passed long ago at the time we measured hopelessness at follow-up. This could also explain why changes in levels of hopelessness from midlife to the point of diagnosis were unrelated to the risk of being diagnosed with Alzheimer’s disease or any other kind of cognitive impairment.

A more trivial, but seemingly quite plausible explanation is related to statistical reliability; at follow-up we measured hopelessness feelings shortly before screening the participants for cognitive impairment. Especially for the participants who were to become diagnosed with cognitive impairment it could easily be assumed that their responses to a questionnaire would be more inconsistent than for the participants with normal cognition, leading to less precise point estimates for this group. We checked this possibility by comparing the variances in hopelessness scores at follow-up within the groups with no cognitive impairment, mild cognitive impairment, and Alzheimer’s disease; our hypothesis being that worse cognitive function would be associated with higher variability. We found no support for this alternative explanation, at least not as reflected by increased variance in scores in the groups with cognitive impairment. This finding seems to strengthen the alternative explanation for the pattern of results we found; that feelings of hopelessness, decades before a diagnosis can be made and closer in time to common estimations of the time window for probable disease initiation [1], could be involved in the causative mechanisms behind dementia development.

Non-participants shared several characteristics at baseline that have been found to predict dementia and they also had higher levels of hopelessness. According to linked register data, a higher proportion of the non-participants also had a dementia diagnosis at the time of follow-up. It therefore seems likely that selective drop out at least did not contribute to over estimate the associations we found.

### Strengths and Limitations

The strengths of the current study include a large, well-characterized and representative population-based sample, a high participation rate (82–90%) at baseline, long follow-up, and careful assessment for cognitive impairment. Access to baseline data and linked registers for non-participants allowed us to evaluate the likelihood of participation bias. Data on a large number of variables from midlife made it possible to adjust the associations between hopelessness and cognitive health for many potential cofounders and also to evaluate the role in these association of ApoE4, the major genetic risk factor for Alzheimer’s disease. The measurement of hopelessness feelings both at baseline and follow-up, in combination with the relatively young participant age at baseline, were important features in order to address the debated question of a prodromal versus a causal relationship between depressive feelings and dementia.

We were able to adjust the associations between hopelessness and cognitive impairment also for global depressive feelings at follow-up. However, the lack of baseline measures of global depression limited our ability to make a full estimation of the specific relevance of hopelessness feelings in relation to other dimensions of depression. We imputed CES-D scores for the 219 participants who lacked data on depression at follow-up, but we also made separate calculations without these 219 persons, i.e. without imputation, and could see that the imputation did not alter the results in any significant way.

Another limitation of the study is that we did not screen for possible cognitive impairment and dementia already at baseline, which potentially could mean that some of our participants, in spite of their young age, were affected by a beginning cognitive impairment disease already when they entered the study. In the following, this possibility will therefore be discussed more in detail.

### The threat of reverse causation

Given the relatively young age of our participants (mean 50.4 years at baseline), the prevalence of dementia should have been very low (>0.1%) at baseline. Survival time after the incidence of dementia has been reported to be between 3.3 [31] and 9.3 years [32]. With a follow-up averagely 21 years after the baseline examinations, it thus seems justified to at least exclude the possibility that among our participants anyone would have fulfilled the diagnostic criteria for dementia already at baseline.

Still, we cannot completely rule out the possibility that some persons at baseline had dementia in a very early, preclinical phase. As age is one of the strongest risk factors for dementia, persons with subclinical dementia already at baseline, if they at all existed, could be expected primarily among participants who at baseline belonged to the upper part of the 34–64 years age range. The separate logistic regression for the relatively younger and older participants at baseline did however not support this possibility.

If feelings of hopelessness are assumed to be prodromal to the disease, it seems reasonable to assume such feelings to become progressively more pronounced as the underlying disease progresses. If this assumption is valid, the absence of any significant increase in hopelessness feelings among those who developed any type of cognitive impairment also speaks against reverse causation as an alternative explanation. In addition, late-life levels of hopelessness were not significantly associated with cognitive impairment, while the associations from baseline were robust.

In summary, our data seem more consistent with the explanation that feelings of hopelessness could constitute an early risk factor for cognitive impairment, rather than reflecting prodromal symptoms.

### Hopelessness versus depression

Although convincing evidence exists that depression can have detrimental effects on cognition per se [33]—and that these effects may be mediated by depression-related changes in hippocampal structure and function [34], doubts have been raised that these changes are related to Alzheimer pathology [10,35–37]. That immunology could be an important link behind the association is indicated by the common feature of neuroinflammaton in both depression and Alzheimer’s disease [38]. Concerning hopelessness, data from our study can neither exclude or support any of these suggested mechanisms [39].

Other possible mechanisms include differences in life style factors between individuals, such as education, diet, alcohol, smoking or physical exercise, that could accompany differences in feelings of hopelessness. We checked several life style indicators, but when we adjusted for those that where associated with feelings of hopelessness, the association still remained. In spite of this, residual confounding cannot be completely excluded.

Previous studies have indicated that feelings of hopelessness could have a special relevance for health, even compared to composite measures of depression [20–22]. Although the association between hopelessness and cognitive impairment remained significant also after adjustment for depression in later life in our study, without data on composite depressive feelings at baseline, we could not make a direct comparison between depressive feelings and feelings of hopelessness from baseline.

At least three other studies have evaluated changes in depressive feelings up to the point of dementia diagnosis. The results of two of them point in a similar direction as our results for hopelessness: baseline levels of depressive feelings predicted cognitive health at least as well as levels proximate to the diagnosis, with barely any increases in levels during the follow-up time [12,13]. In contrast to the third study [14], our results suggest a similar association also with Alzheimer’s disease, at least for feelings of hopelessness.

### The role of ApoE4

Previous studies suggest that the ApoE4 allele is not only a risk factor in itself for Alzheimer’s disease [40], but also can make its carrier more vulnerable to other risk factors, [41,42] including depression [43]. Our results indicate that this is also true for feelings of hopelessness. On the other hand, hopelessness [20–22] and other negative feelings, such as grief [44] and composite depressive feelings [45] are all risk factors for a range of other diseases. Taken together, this might mean that carrying negative feelings during the life course is not a specific risk factor for Alzheimer’s disease, but rather that Alzheimer’s disease could be the more probable disease outcome from such exposure for persons who are also ApoE4 carriers.

## Conclusion

Feelings of hopelessness already in midlife may have long-term implications for cognitive health with a pronounced risk increase for Alzheimer’s disease in persons who in addition carry the ApoE4 allele. While most research on emotion and cognitive health has focused on global depression or composite depressive feelings, this study suggests a more differentiated approach to be feasible. The dimension of hopelessness seems like a promising candidate that may have relevance beyond that of global depression—also as a risk factor for Alzheimer’s disease and other dementias.
