# Correction: Feelings of Hopelessness in Midlife and Cognitive Health in Later Life: A Prospective Population-Based Cohort Study

**DOI:** 10.1371/journal.pone.0142465

**Published:** 2015-11-04

**Authors:** Krister Håkansson, Hilkka Soininen, Bengt Winblad, Miia Kivipelto

The images for Figs 2 and 3 are incorrectly switched. The image that appears as Fig 2 should be Fig 3, and the image that appears as Fig 3 should be Fig 2. The figure captions appear in the correct order. Please view the correct Figs 2 and 3 here.

**Fig 2:**
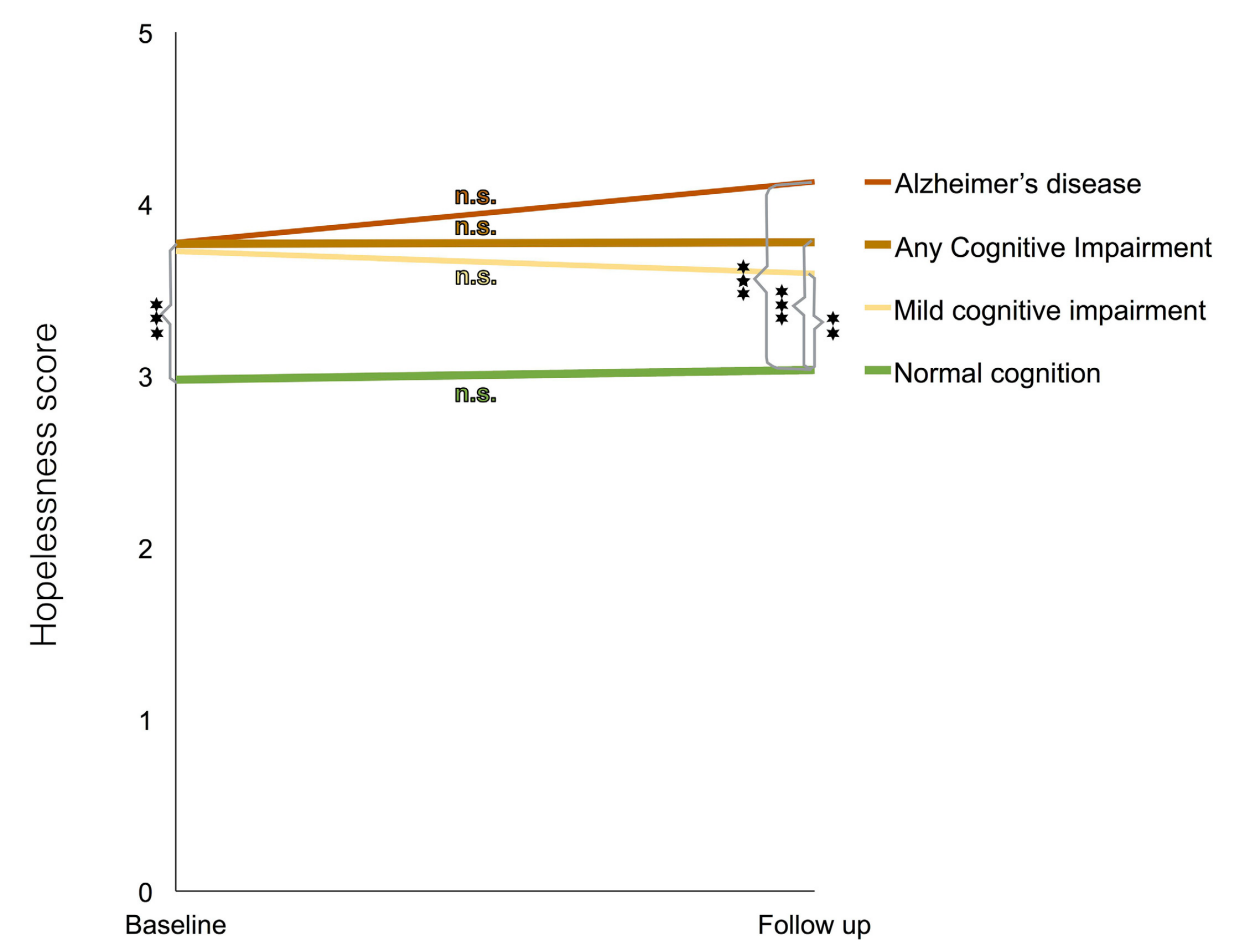
Levels of hopelessness at baseline and follow-up for the different outcome groups. Changes in hopelessness scores from baseline until follow up for those who at follow-up were either without cognitive impairment or were diagnosed with any cognitive impairment, mild cognitive impairment or Alzheimer’s disease. No changes from baseline to follow-up were statistically significant within any of the outcome categories (Student t-test for independent samples). All differences between the non-impaired group and any of the cognitive impairment groups were statistically significant, both at baseline and at follow-up (* = p≤10.05, ** = p≤0.01, *** = p≤0.001 as indicated by Students t-test for paired samples). The graph is based on scores from participants with measurements of hopelessness both at baseline and follow-up (N = 1246).

**Fig 3:**
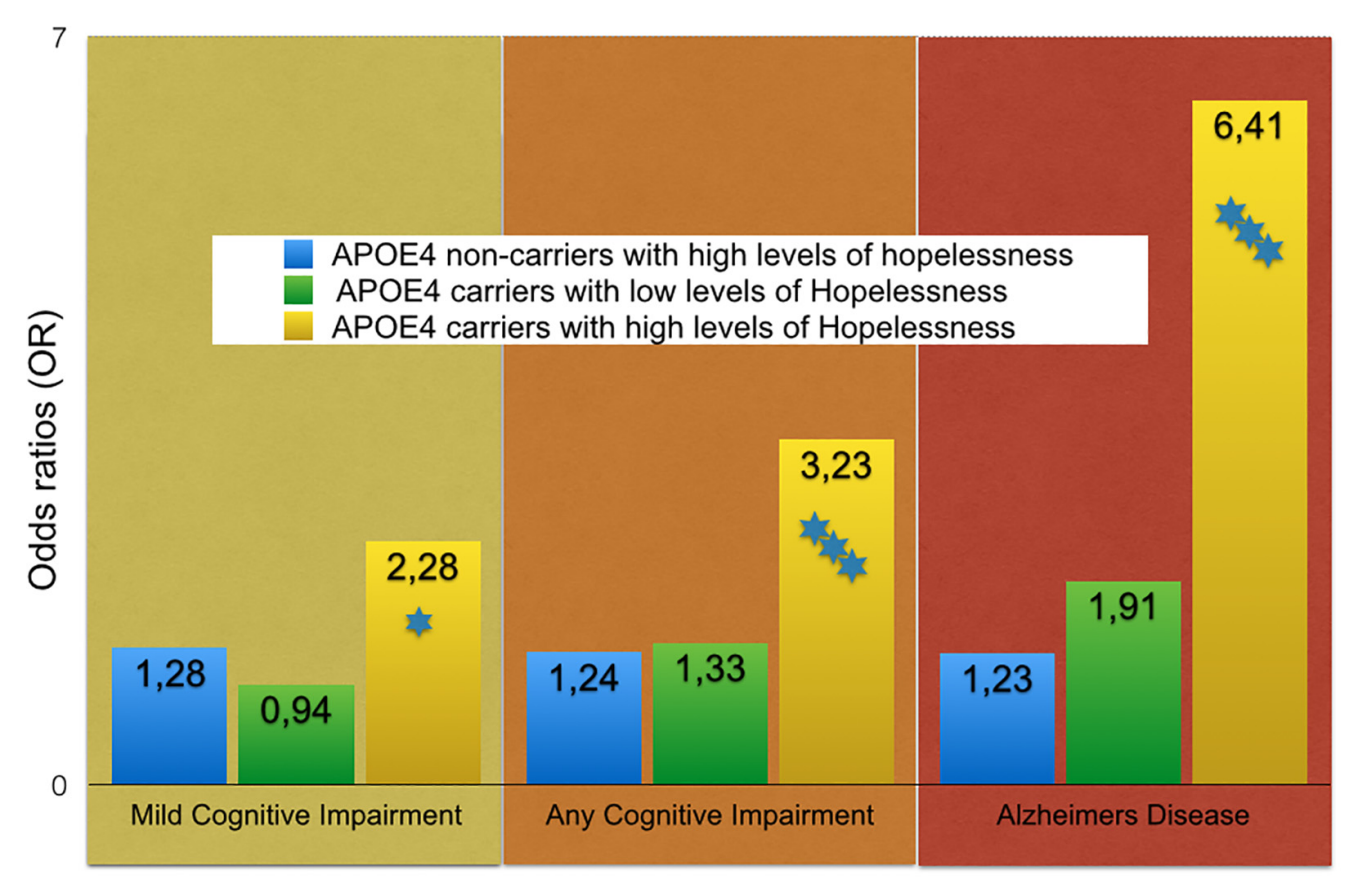
Hopelessness and cognitive impairment for ApoE4 carriers and non-carriers. Odds ratios from logistic regressions after adjustments for age, education, gender, BMI, blood pressure, cholesterol, residence area, occupation, physical activity, smoking, marital status, and depression. The risk is in comparison to ApoE4 non-carriers with low levels of hopelessness at midlife (OR = 1). Stars indicate the level of statistical significance (three levels <0.05, <0.01, <0.001).
